# Molecular and Phylogenetic analysis revealed new genotypes of *Theileria annulata* parasites from India

**DOI:** 10.1186/s13071-015-1075-z

**Published:** 2015-09-17

**Authors:** Neena George, Vasundhra Bhandari, D. Peddi Reddy, Paresh Sharma

**Affiliations:** National Institute of Animal Biotechnology-DBT (NIAB), D. No. 1-121/1, 4th and 5th Floors, Axis Clinicals Building, Miyapur, Hyderabad, Telangana India

**Keywords:** *Theileria annulata*, 18S rRNA, Genotype, Diversity, India

## Abstract

**Background:**

Tick borne diseases impinge cattle worldwide causing mortality and resulting in huge economic losses. Theileriosis is one of the important tick borne diseases mainly caused by *Theileria annulata* and one of the commonly occurring infections among the livestock. *T. annulata* causes immense loss to the livestock industry and therefore, efficacious eradication and control strategies are needed for the control of the disease. Genetic diversity among *T. annulata* parasites is another important aspect which is overlooked in India. Thus, the present study aims to evaluate the prevalence along with genetic diversity and phylogeny of the prevailing *T. annulata* population of India.

**Methods:**

Genomic DNA was extracted from cattle blood samples (*n* = 862) from different regions of Andhra Pradesh. Molecular diagnosis using *T. annulata* 18S rRNA based PCR was performed to detect parasites in cattle. Further, 18S rRNA gene was cloned and sequenced to determine similarity and diversity from the known *T. annulata* sequences.

**Results:**

We observed an overall prevalence rate of 32.40 % *T. annulata* infection in Andhra Pradesh based on PCR assay. The sequence analysis revealed novel genotypes among the *T. annulata* strains from India. Thirteen strains showed closed proximity with a strain from China whereas one Indian strain showed similarity with a South African strain [*Theileria sp* (buffalo)] based on phylogenetic analysis. Nucleotide heterogeneity of the 18S rRNA sequence among the strains examined varied from 0.1 to 8.6 % when compared with the published strains.

**Conclusion:**

The present study provides us with the molecular prevalence of theileriosis, and will support the accomplishment of actions or in design of strategy to control theileriosis transmission to cattle. Additionally, it highlights the emergence of strains with novel genotypes from India.

## Background

Theileriosis is a tick borne disease which results in mortality and economic loss amounting to US$ 800million approximately in India [1]. It is a lymphoproliferative disease caused by an apicomplexan intracellular parasite, *Theileria annulata* in cattle and water buffaloes. The clinical symptoms comprise of fever, anaemia and jaundice and the disease is fatal, if left untreated [2]. Control of infection is mainly banking on an attenuated vaccine which is not effective and has problems like cold chain and short-term protection. An additional problem associated with the disease is high treatment cost not bearable by farmers, emerging drug resistance and lack of sufficient studies focussed on epidemiology and prevalence of theileriosis in India. During theileriosis recovery, primary infection may result in the development of a carrier state or relapse case and further, these carrier animals play a crucial role in the maintenance of the life cycle of the parasite by the ticks.

Diagnosis of the disease based on microscopic examination of Giemsa stained blood smears and lymph node biopsy is disadvantageous in case of low parasitemia and carrier state animals [3, 4]. However, the molecular based assays such as PCR have proven to be the most reliable tool for detecting *Theileria* parasites in clinical and subclinical cases of theileriosis especially detecting carrier state [3–7]. A number of control measures have been employed such as acaricides application, chemotherapy and vaccination, however, due to lack of knowledge on the prevalence of *Theileria* infection these measure are not effective [8, 9].

In addition to that, studies investigating the genotypic diversity among *T. annulata* parasites from India are scarce [10, 11]. The role of 18S rRNA gene in *Theileria* genetic diversity studies holds importance due to the presence of a conserved and a hypervariable region (V4) which are well studied and crucial for determining evolutionary patterns and similarity among the *Theileria* species [12, 13]. Moreover, it has been sequenced from a large number of strains belonging to different parts of the globe and database set is readily available for comparison [11, 12, 14–18]. In India, there is a need for studies focusing on the prevalence of theileriosis and to identify genetic diversity among *T. annulata* strains to make the control programs effective and successful. In the present study, we have randomly collected samples from the state of Andhra Pradesh (now divided into Seemandhra and Telangana) focusing on the epidemiology, molecular characterization of *T. annulata* parasite and to investigate genotypic variations among the strains.

## Methods

### Ethics statement

The study involved drawing of ~ 5 ml blood from jugular vein aseptically from cattle with the consent of the farm owners. There is no specific law for blood sample collection and hence no approval was mandatory.

### Sample collection and DNA isolation

Blood samples were collected from 15 districts of Andhra Pradesh in K2 EDTA vacutainer tube (BD, Franklin, USA) as mentioned in Table 1. Samples were collected randomly during the period of November 2013 to March 2015 from native and cross breed cattle from the jugular vein directly into vacutainer tubes using BD Vacutainer Eclipse Blood Collection Needle (BD, Franklin, USA). DNA was isolated from the blood samples using QIAamp DNA Mini Kit as per manufacturer’s instructions (Qiagen). The quality and quantity of the DNA were determined using Nano drop and gel electrophoresis. Samples were stored at −80 °C until further use.

**Table 1:** Details of blood sample collected from different districts of Andhra Pradesh

S. No	District name	Total samples	PCR positive	Prevalence rate (%)
1	Adilabad	53	27	50.90
2	Karim Nagar	48	21	43.80
3	Nizamabad	41	5	12.20
4	Warangal	42	20	47.60
5	Medak	139	78	56.10
6	Hyderabad	75	18	24.0
7	Rangareddy	66	23	34.8
8	Nalgonda	57	9	15.80
9	Anantpur	40	19	47.5
10	Chittoor	73	9	12.30
11	Guntur	50	0	0
12	Krishna	45	7	15.56
13	Srikakulam	49	14	28.57
14	Visakhapatnam	45	3	6.67
15	Vijayanagaram	39	26	66.67
	Total Samples	862	279	32.4

Foot note: District from serial number 1–8 in the table belongs to Telangana while serial number 9 to 15 belongs to Seemandhra

### Culture of *T annulata* parasite and genomic DNA isolation

*T. annulata* infected macrophages were isolated from the clinically infected cattle and culture was maintained at 37 °C in RPMI-1640 medium supplemented with 25 mM HEPES, 4 mM L-glutamine, 100 μg/mL penicillin, 100 μg/mL streptomycin and 10 % Foetal bovine serum (FBS) with 5 % CO_2_ [19]. Parasite enrichment was done from the *T. annulata* infected macrophages cell line using percoll gradient as per Langsley *et al.* [19] with slight modifications. DNA was isolated from the enriched parasites using QIAamp DNA Mini Kit as per manufacturer’s instructions (Qiagen).

### PCR Amplification of 18S rRNA gene of *T. annulata*

PCR was used to amplify 125 bp fragment of *T. annulata* 18S rRNA gene [Genbank:EU083801.1] with species-specific primers, Forward (5′- ACGACTCCTTCAGCACCTTG-3′) and Reverse (5′-AAATTAAGCCGCAGCTCCAC-3′). PCR reactions were performed using Speed Star HS DNA polymerase (Takara) in an automatic DNA thermocycler (Biorad-T100 Thermal cycler). Reactions were performed in 25 μl volumes with 100 ng of purified genomic DNA. PCR conditions consisted of a first denaturation step of 1 min at 95 °C, followed by a second step of 35 cycles of 10 s at 95 °C, 20s at 61 °C and 10 s at 72 °C, and a final extension step of 1 min at 72 °C. DNA extracted from *in vitro* cultured *T. annulata* parasite was used as positive whereas distilled water and *T. oreintalis* positive DNA sample were used as negative controls, respectively in all PCR reactions. PCR products were separated by 2 % agarose gel electrophoresis and stained with ethidium bromide to assess the presence of specific bands indicative of *T. annulata* . Gels were photographed under gel documentation system (Syngene) and the size of each PCR product determined by reference to a DNA ladder (NEB).

### Senstivity and specificity of 18S rRNA based PCR

Ten fold serial dilution of plasmid cloned 18S rRNA gene was prepared and further, utilized to evaluate the sensitivity of the PCR using conditions as described above. The highest dilution of the DNA showing a visible band was taken as the detection limit. Specificity of primer was also determined by using DNA samples positive for *T. orientalis* species infection.

### Cloning and sequencing of 18S rRNA gene

One representative positive PCR sample (*n* = 14) from each district was selected for 18S rRNA gene cloning. The gene was amplified using primers (18S rRNA Forward: GGC CAG TAG TCA TAT GCT TGT and Reverse: TGA TCC TTC CGC AGG TTC ACC) and further, the amplified PCR products (base pair (bp) = 1728) were purified by Nucleospin Gel and PCR cleanup kit (Macherey Nagel, Germany) following the manufacturer’s instructions. After purification, 1728 bp PCR product was cloned into a TOPO cloning vector (Invitrogen, Life Technologies) followed by transformation into Top10 cells with ampicillin as a marker. Positive colonies were selected by PCR followed by restriction digestion with *Eco*R1 enzyme. Plasmid from positive colonies were isolated using Nucleospin plasmid kit (Macherey Nagel, Germany) and sequencing (five clones per sample) was performed using universal primers of M13 gene present on the backbone of the TOPO vector. Nucleotide sequence of the 18S rRNA gene was determined by DNA sequencing sent to a commercial laboratory by Sangers sequencing. Sequences were compared with 18S rRNA gene sequences of *T. annulata* parasites available in the NCBI database using the Basic Local Alignment Search Tool (BLAST) followed by multiple alignment using ClustalW2. The representative sequences obtained were registered in the NCBI GenBank nucleotide sequence database under the assigned accession numbers from KT367866 to KT367879 which corresponds to TA 1 – TA 14.

### Phylogenetic tree and multiple alignment using 18S rRNA gene

Multiple alignment was done using ClustalW2 alignment method using 18S rRNA gene sequences. The evolutionary history was inferred by using the Maximum Likelihood method based on the Kimura 2-parameter model [20]. The tree with the highest log likelihood (−25870.0411) is shown. The percentage of trees in which the associated taxa clustered together is shown next to the branches. Initial tree(s) for the heuristic search were obtained automatically by applying Neighbor-Join and BioNJ algorithms to a matrix of pairwise distances estimated using the Maximum Composite Likelihood (MCL) approach, and then selecting the topology with superior log likelihood value. The tree is drawn to scale, with branch lengths measured in the number of substitutions per site. The analysis involved 42 nucleotide sequences. All positions containing gaps and missing data were eliminated. There were a total of 965 positions in the final dataset. Evolutionary analyses were conducted in MEGA6 [21].

### Sequence distances

Divergence within the 18S rRNA gene sequenced from different districts was calculated in MEGA6 by comparing with the other available 18S rRNA sequences of *T. annulata* from NCBI. Divergence is calculated by comparing sequence pairs in relation to the phylogeny reconstructed by MEGA6. MEGA6 calculates the divergence between two branches by comparing sequence pairs in relation to the reconstructed phylogeny. Total Distance is the sum of all branch lengths. Distance (i,j) = sum (residue distances) + (gaps × gap penalty) + (gap residues × gap length penalty). When a direct pathway exists between two sequences on the tree, Distance (i,j) equals the sum of the branch lengths. Divergence (i,j) = 100 [Distance (i,j)]/Total Distance.

### Statistical analysis

Confidence interval (CI) at 95 % was calculated using online software (https://www.mccallum-layton.co.uk/tools/statistic-calculators/confidence-interval-for-proportions-calculator).

## Results

### Molecular prevalence

**Fig. 1:**
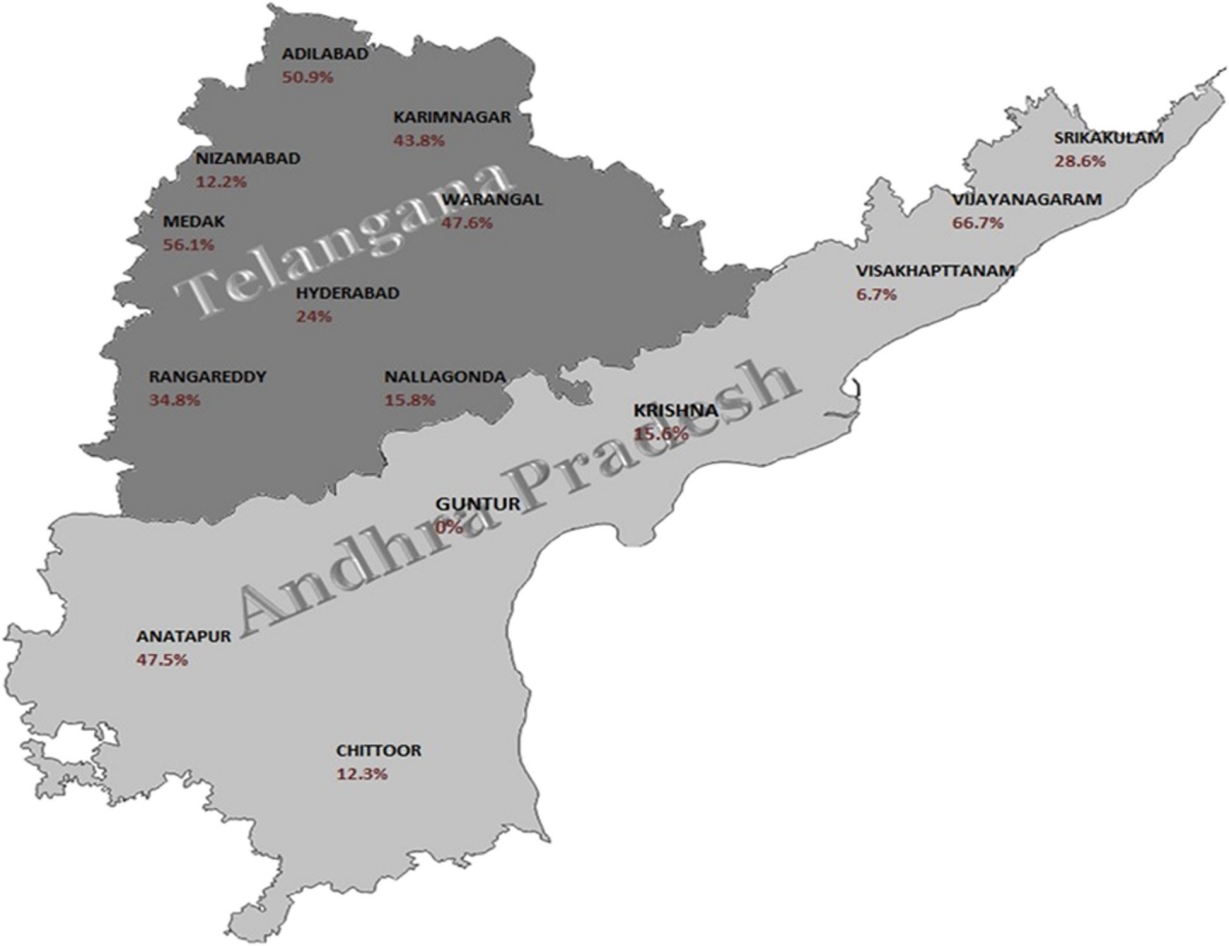
Outline map of Andhra Pradesh. 15 districts from where samples are collected are indicated and below each district the prevalence rate (%) is mentioned in parenthesis

**Fig. 2:**
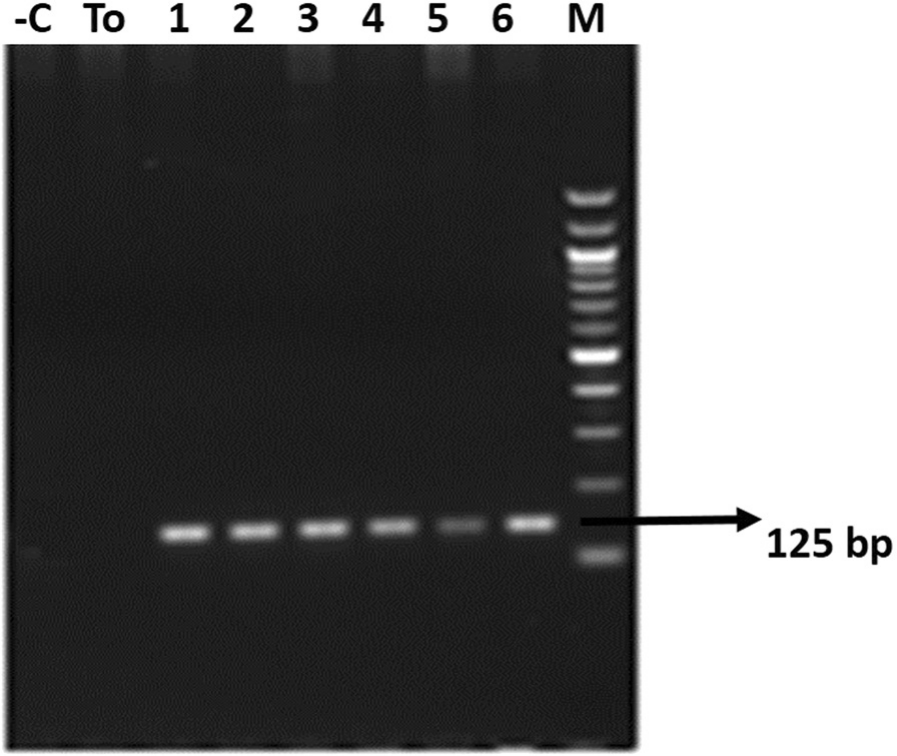
PCR amplification in cattle DNA samples. Agarose gel electrophoresis of amplified DNA from different cattle blood DNA samples by using 18S rRNA. Lanes: 1-Negative control distill water: Lane 2-*T. orientalis* positive DNA sample; Lane 3 to Lane 8 positive blood DNA samples from cattle; Lane 9–100 base pairs DNA ladder

**Fig. 3:**
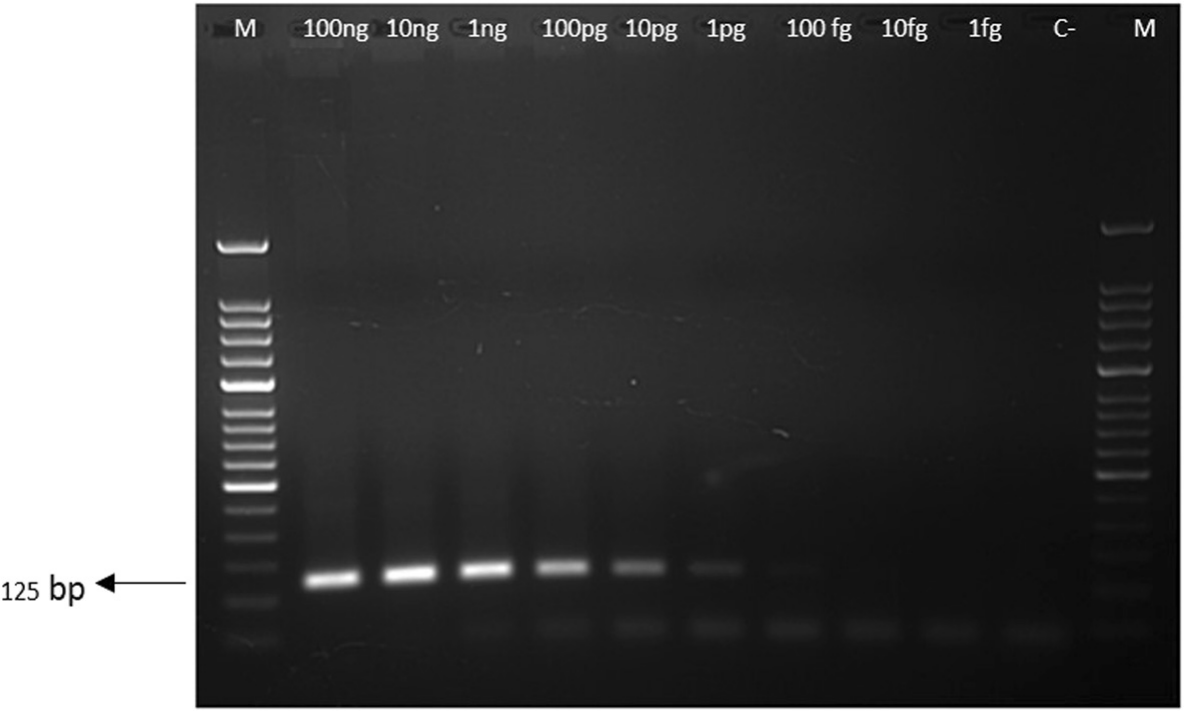
Sensitivity of 18S rRNA primer. Agarose gel electrophoresis of amplified DNA from serially diluted plasmid cloned 18S rRNA gene by using 18S rRNA. Lane 1: 100 ng DNA; 2: 10 ng; 2: 1 ng; 4: 100 pg; 5: 10 pg; 6: 1 pg & Lane 7: 50 base pairs DNA ladder

We have collected a total of 862 cattle blood samples out of which 521 samples belong to Telangana and 341 to Seemandhra (Fig. 1). 18S rRNA primer was used to detect the presence of *T. annulata* infection in cattle and DNA isolated from *T. annulata* cell line was used as positive control. PCR amplification from blood DNA of cattle corresponding to band size of 125 bp were considered as positive for *T. annulata* (Fig. 2). Further, primer sensitivity was also analyzed using plasmid cloned 18S rRNA and it was able to detect till 1picograms of DNA (Fig. 3). Andhra Pradesh is divided into 22 districts out of which we collected samples from a total of 15 districts, 8 districts belonging to Telangana and 7 from Seemandhra, with a minimum of 39 samples from each as mentioned in Table 1 and Fig. 1. 201 samples were positive out of 521 in Telangana amounting to 38.60 % prevalence rate of *T. annulata* infection whereas in Seemandhra the prevalence rate was lower to 22.90 % with only 78 cases to be positive out of 341. Of the 8 Telangana districts studied, prevalence rate ranged from 12.20 to 56.10 % while in 7 districts of Seemandhra, it ranged from 0 to 66.67 %. Overall, prevalence rate in both the adjoining state (Telangana and Seemandhra) is 32.40 % at 95 % CI of ±3.2 %.

### Genetic diversity and phylogenetic analysis

**Fig. 4:**
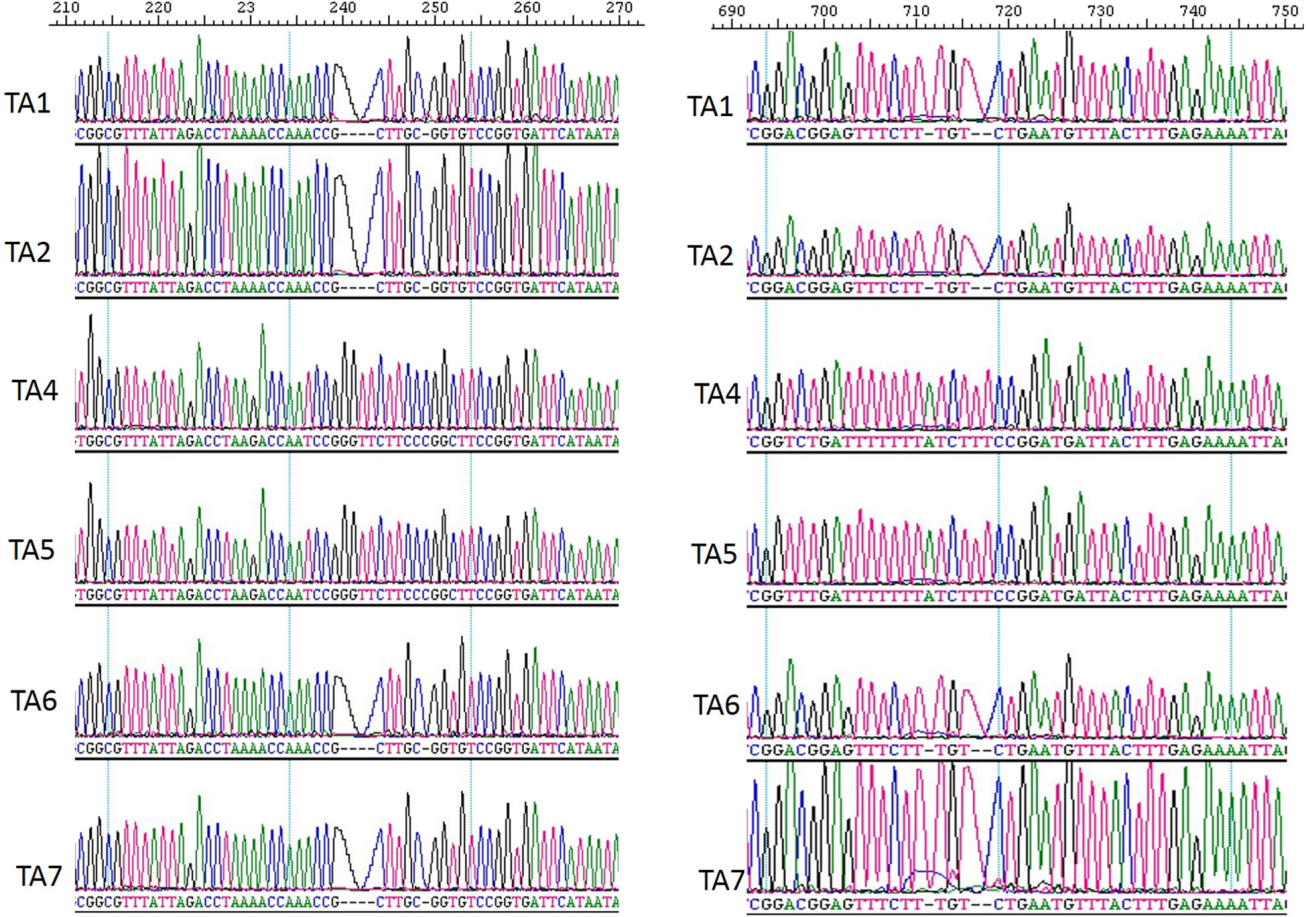
Novel insertions in 18S rRNA gene of *T. annulata* strains from India. Sequence chromatograms from TA 1, TA 2, TA 4, TA 5, TA 6, TA 7 showing the insertions in the TA 4 and TA 5 sequence. Nucleotide position is based on the Iran strain [GenBank:KF429795] SSU rRNA gene sequence

18S rRNA gene (1728 bp) was amplified from DNA of one representative strain from each district followed by cloning and sequencing. Multiple alignment and sequence analysis of the 18S rRNA gene identified novel SNPs in the fourteen isolates by using Iran vaccine strain [GenBank:KF429795] as the reference. Gene sequence of the previously reported Indian strains were not used as reference, due to non-availability of complete gene sequence. However, during SNPs analysis, SNPs in common with previously sequenced Indian strains were not included in the results. SNPs were noted from both hypervariale and the conserved region of the gene. A total of 88 SNPs were identifed during the analysis. 63 SNPs were present commonly between TA 4 and TA 5 while TA 5 had an additional 4 extra SNPs in its sequence as compared to another 12 strains. TA 4 and TA 5 strains also showed novel insertions at positions; 240–243, 249, 293, 712, 716 and 717 in comparison to the reference gene sequence (Fig. 4).

**Fig. 5:**
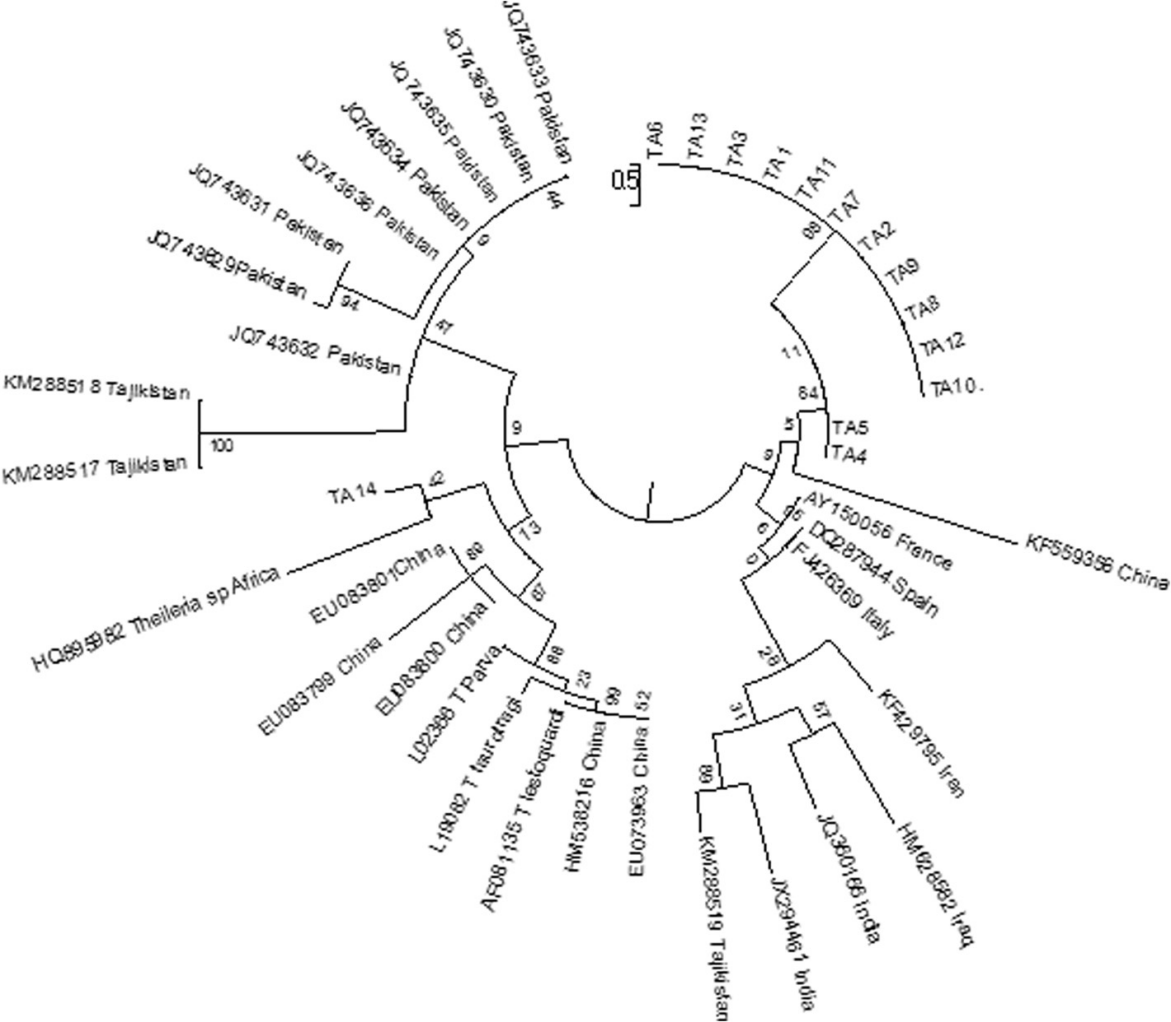
Phylogenetic analysis. Evolutionary analysis of the 18S rRNA gene showing the phylogenetic relationship of the *Theileria annulata* (TA 1- TA 14) with other known *Theileria* species. Phylogenetic analysis was performed using MEGA6 software. Branch lengths are proportional to the estimated genetic distance between the species. GenBank accession numbers are indicated in parentheses in the figure. Fourteen representative DNA samples of each district were included in the analysis: TA 1 from Krishna district [GenBank: KT367866], TA 2 from Anantapur [GenBank: KT367867], TA 3 from Adilabad [GenBank: KT367868], TA 4 Medak [GenBank: KT367869], TA 5 from Karimnagar [GenBank:KT367870], TA 6 from Nizamabad [GenBank:KT367871], TA 7 from Hyderabad [GenBank:KT367872], TA 8 from Warangal [GenBank: KT367873], TA 9 from Rangareddy [GenBank: KT367874], TA 10 from Nallagonda [GenBank:KT367875], TA 11 from Chittor [GenBank: KT367876], TA 12 from Visakhapatnam [GenBank:KT367877], TA 13 from Srikakulam [GenBank:KT367878], TA 14 from Vijayanagaram [GenBank: KT367879] districts from Andhra pradesh and Telangana

Multiple alignment was performed with 14 sequences representative of each district [TA 1-TA 14] and 13 sequences of *T. annulata* parasite strains available in the GenBank database using the MEGA6 method. Also, 18S rRNA gene sequences of leucocyte transforming *Theileria* parasites like *T. parva* [L02366], *T. taurotragi* [L19082], *T. lestoquardi* [AF081135] and *Theileria* sp. Africa [HQ895982] other than *T.annulata* were also included in phylogenetic analysis. Phylogenetic tree clustered the 18S rRNA sequences from the 14 strains into two groups with one large sub group containing sequences from all the parasite strains,TA 1-TA 13 along with a strain from China [GenBank:KF559356] (Fig. 5). Further, there is a subclustering within this branch for TA 4 and TA 5 strain. The second group containing the TA 14 showed clustering with the *Theileria* (buffalo) species reported to cause corridor disease in South Africa (Fig. 5). In order to examine the variations among the 14 strains and their clustering partners, divergence analysis was carried out. Divergence among the the 18S rRNA sequences ranged from 0.1 to 8.6 %. Sequence homology of strains TA 4 and TA 5 was found to be 91.9 % in comparison to previously reported Indian strain [GenBank:JX294461]. Sequence variation was high between reference sequence and the strains. Whereas, exceptional sequence similarity of 98.26 % was found between TA 14 and the South African strain.

## Discussion

There are number of hemo-protozoan parasites infections in cattle out of which *T.annulata* accounts for maximum infections [22]. The disease causes economic loss due to high mortality, reduction in milk yield, emaciation as well as an increased burden due to treatment cost to the farmer [1]. Further, impeding the problem is the lack of information available about the prevalence rate of the infection with the consequence of poor implementation of control policy.

In our study, we found high prevalence rate of 32.40 % for *T. annulata* infections in cattle at Andhra Pradesh by utilizing 18S rRNA based PCR. The samples were randomly collected from cattle, either cross breed or native breed from different districts of Andhra Pradesh. In India, there are few reports with less numbers showing the presence of *T. annulata* infection using molecular based assay for e.g., a study from Gujarat where 74 samples were detected positive out of 113 [4], in Bangalore 41 cases were positive out of 132 [23] while a study from Punjab showed a prevalence rate of 14.65 % using microscopic examination [22]. The current finding will help to shed light on the occurrence of *T. annulata* infection in Andhra Pradesh and bring it to the forefront of devising control policy to detect and eliminate the infection resulting in reducing the economic losses caused due to the disease.

Further, we have performed phylogenetic analysis and multiple alignment of the prevailing *T. annulata* strain population from Andhra Pradesh (Telangana and Seemandhra) using 18S rRNA gene. Multiple alignment revealed TA 4 & TA 5 strains with novel insertions (*n* = 9) and SNPs clustering with a strain from China [GenBank:KF559356] which may be due to evolutionary changes and might be important for adaptation. Further, maximum number of strains [TA 1-TA 13] showed proximity to China whereas TA14 to South African *Theileria (buffalo)* species [Genbank:HQ895982], however, forming a separate subclade on the evolutionary tree [4]. The sequence similarity indicates that it might be due to bi-directional transfer or as a result of evolutionary changes. Similarity of TA 14 with South African strain raises concern since, South African strain is reported to be potentially lethal in cattle [24].

Reported diversity studies of *T. annulata* strains had considered the complete gene sequence or only the hypervariable region, V4 (~200 bp) of the 18S rRNA gene [9, 10, 12–15, 25]. In the current study, novel SNPs were found in all fourteen strains in the conserved as well as the hypervariable region indicating presence of new genotypes of *T. annulata* strain from India. TA 4 and TA 5 strains showed maximum changes in their nucleotide composition in comparison with the published sequence of Iran strain [GenBank:KF429795]. The study showed the presence of genotypes which cluster with the known *T. annulata* strains and other leukocyte transforming *Theileria* strains, however, forming separate sub-clades with 0.1 to a maximum 8.6 % nucleotide heterogeneity. Our data clearly show that *Theileria* genotypes detected are clearly distinct from earlier reported strains of India.

Overall, our study indicates the molecular prevalence of theileriosis and identification of novel genotypes of *T. annulata* strains in Andhra Pradesh, India.

## Conclusion

The current study reveals a prevalence rate of about 32.40 % in the states of Andhra Pradesh and Telangana. It is a first step towards representing the occurrence of the *T. annulata* infection in this region. It will possibly raise the alarm for effective and urgent measures against the disease control as well as identification of novel genotypes of *T. annulata* in the prevailing population.
